# A high-capacity model for one shot association learning in the brain

**DOI:** 10.3389/fncom.2014.00140

**Published:** 2014-11-07

**Authors:** Hafsteinn Einarsson, Johannes Lengler, Angelika Steger

**Affiliations:** ^1^Department of Computer Science, Institute of Theoretical Computer Science, ETH ZürichZürich, Switzerland; ^2^Collegium HelveticumZürich, Switzerland

**Keywords:** one shot learning, hetero-associative memory, relation learning, bootstrap percolation, iterative retrieval, stochastic Hebbian learning, memory capacity

## Abstract

We present a high-capacity model for one-shot association learning (hetero-associative memory) in sparse networks. We assume that basic patterns are pre-learned in networks and associations between two patterns are presented only once and have to be learned immediately. The model is a combination of an Amit-Fusi like network sparsely connected to a Willshaw type network. The learning procedure is palimpsest and comes from earlier work on one-shot pattern learning. However, in our setup we can enhance the capacity of the network by iterative retrieval. This yields a model for sparse brain-like networks in which populations of a few thousand neurons are capable of learning hundreds of associations even if they are presented only once. The analysis of the model is based on a novel result by Janson et al. on bootstrap percolation in random graphs.

## 1. Introduction

In the last decades the problem of fast pattern learning has been intensively studied. Amit and Fusi (1994) introduced a model of auto-associative memory for sparsely coded patterns in fully connected neuronal networks and showed that in this model an ensemble of *N* neurons can store almost quadratically many patterns before it starts forgetting old ones, even if each pattern is only presented once. In this paper we consider hetero-associative memory instead of auto-associative memory, i.e., relation learning instead of pattern learning. Moreover, we do not only require fast learning, but also fast retrieval of the learned associations. We incorporate this requirement into our model by considering for each retrieval only the first spike of each neuron, ignoring all further spikes. In particular, our model is spike-based rather than rate-based.

**Figure 1:**
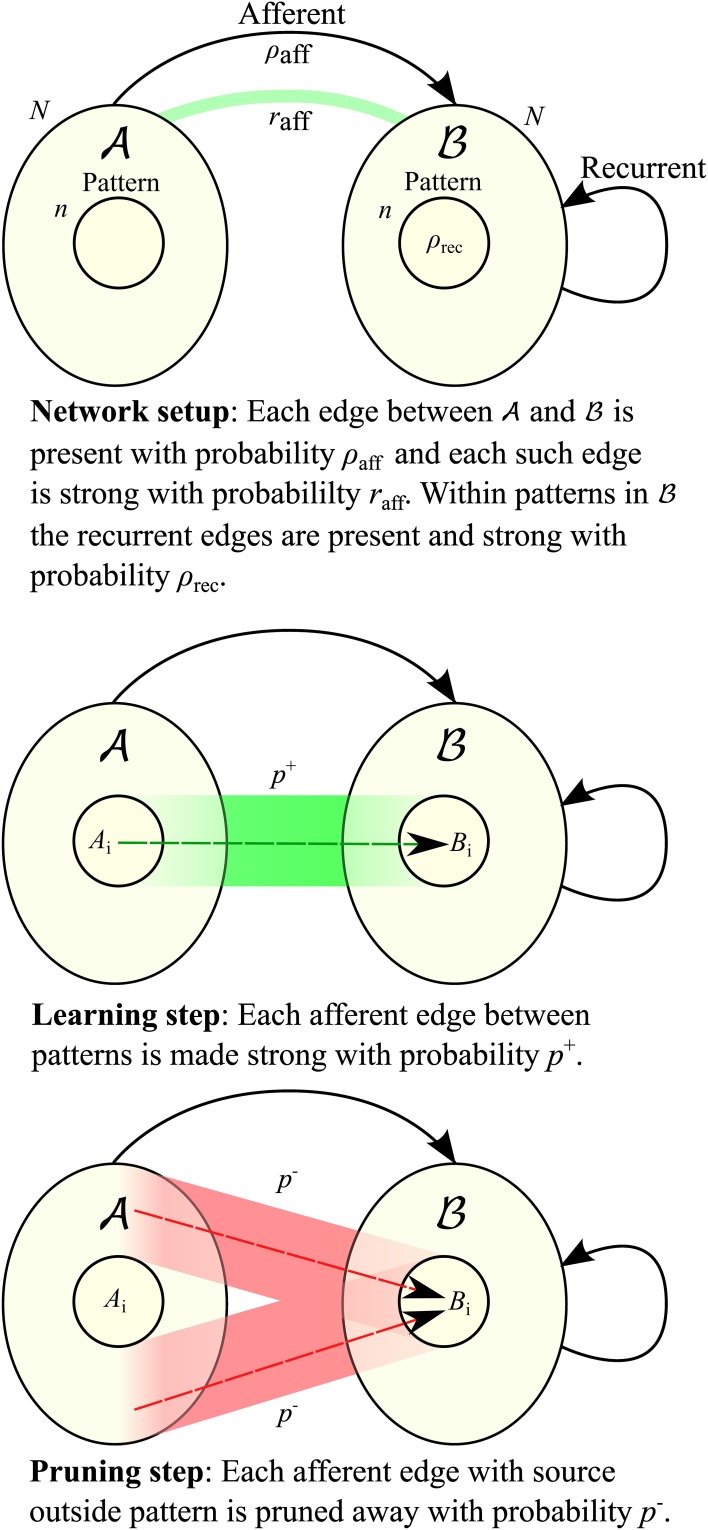
**Network setup and learning procedure**. See Section 2.1 for a more detailed explanation.

Traditionally there have been two main models for hetero-associative memory: the model by Willshaw et al. (1969) based on clipped Hebbian learning, and the networks introduced by Hopfield (1982) (see also Knoblauch et al., 2010 for a review and comparison). Both achieve storage capacities close to the information theoretic upper bound for sparsely coded patterns (Knoblauch et al., 2010). The Hopfield networks are rate-based and aim for convergence to a stable state through auto-feedback, thus they are designed for retrieval in medium or long time scale. The fast learning model in Amit and Fusi (1994) falls in this category, and we compare with it in more detail in Section 2.2. On the other hand, the Willshaw model is both fast-learning and fast-retrieving, but high capacities come at the cost of low retrieval accuracy (Buckingham and Willshaw, 1991). Various ways have been found to overcome this issue, including adaptive thresholds as in Buckingham and Willshaw (1991) and bidirectional iterative retrieval schemes as in Sommer and Palm (1998). Our model is related to the latter approach, except that we are more restrictive in the retrieval procedure so that the model is still fast-retrieving (cf. also Section 4.1): we consider a bipartite graph with partite sets 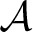 and 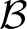, where all edges are directed from 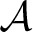 to 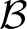 (“afferent edges”), and iterative retrieval is only achieved by the edges in 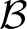 (“recurrent edges”) (see Figure 1 for the setup). In this respect, a similar retrieval scheme for the Willshaw model has been studied by Knoblauch and Palm (2001), with the difference that they used inhibition to stop the spread of activity after the pattern is activated, and that they use a global feedback scheme for threshold control. The latter feature allowed for higher fidelity of retrieval and for a threshold that it is independent of the pattern size. While the Willshaw model may also serve as a model of fast learning, we follow the approach in Amit and Fusi (1994) and use binary Hebbian learning with pruning (see below) so that the total number of synapses is unaffected by the number of learned associations. However, in contrast to the model of Amit and Fusi, since we only consider the first spike of each neuron, a neuron can never go from state “active” to “inactive” since it can not retract a spike that it elicited earlier. All these restrictions are biologically motivated, and the biological background can be found in more detail in Section 4.1.

**Figure 2:**
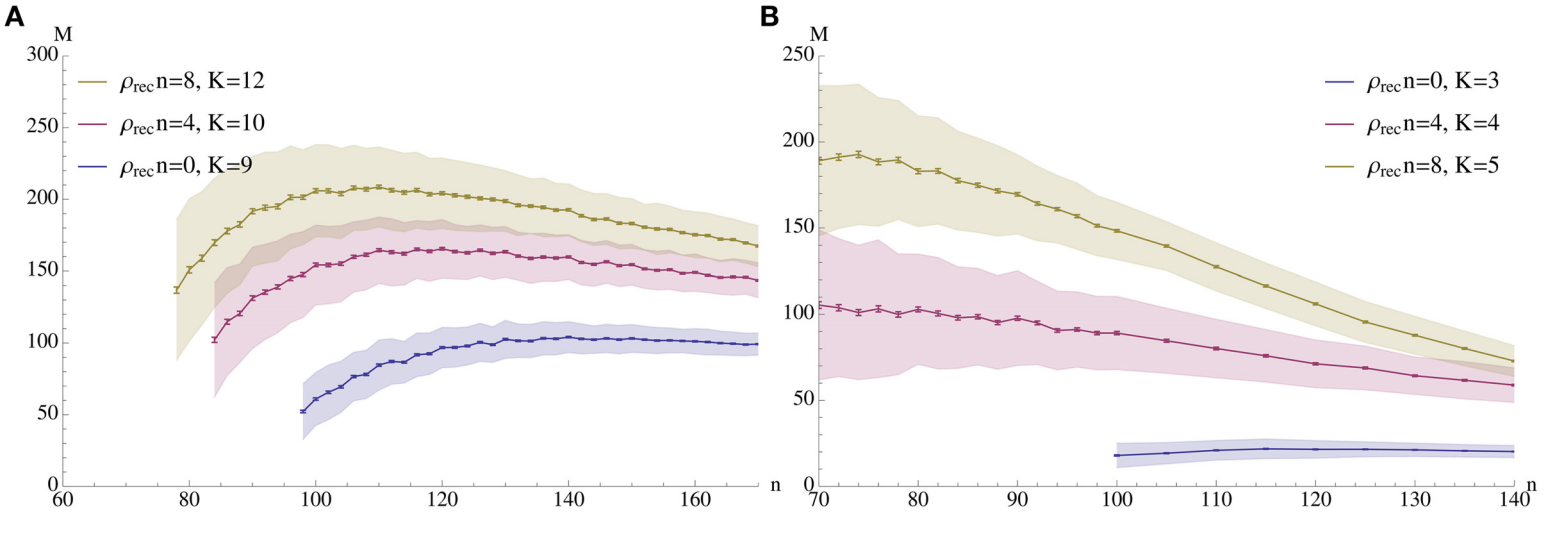
**Capacity (*M*) vs. pattern size (*n*)**. The plots show the mean number of associations that can be inserted until the first association is no longer memorized. The blue curves correspond to a setup without percolation (i.e., recurrent density is 0), while the red and yellow curve correspond to percolation with recurrent degree 4 and 8 respectively. In **(A)** ρ_aff_ = 0.2, *r*_aff_ = 0.1, *p*^+^ = 0.6; the values for *K* were chosen to give stable results for *n* in a wide range. **(B)** Shows a sparser setup with ρ_aff_ = 0.05. Here *r*_aff_ = 0.05, *p*^+^ = 0.6 and *K* is again chosen to optimize capacity. In both experiments *N* = 5000 and the mean is over 500 trials with standard deviation as shown; error bars represent standard mean error. In both cases capacity was only considered if at least 99% of all insertions were successful.

The guiding idea for our model is that a pattern may be stored locally in a cortical column of *N* ≈ 5000 neurons, but that it is necessary to associate patterns in different columns or even different regions of the brain. Therefore, the density between different patterns is much lower than the density within a pattern (Binzegger et al., 2004). The combination of low afferent density and a population size of only 5000 neurons makes it impossible to transfer the existing models for fast learning straightforwardly (cf. Section 2.2). However, by making use of recurrent connections (connections between neurons within a pattern) we are able to show that the resulting iterative retrieval of the pattern allows our model to operate in the range prescribed by biology (see Figure 2 for an example).

**Figure 3:**
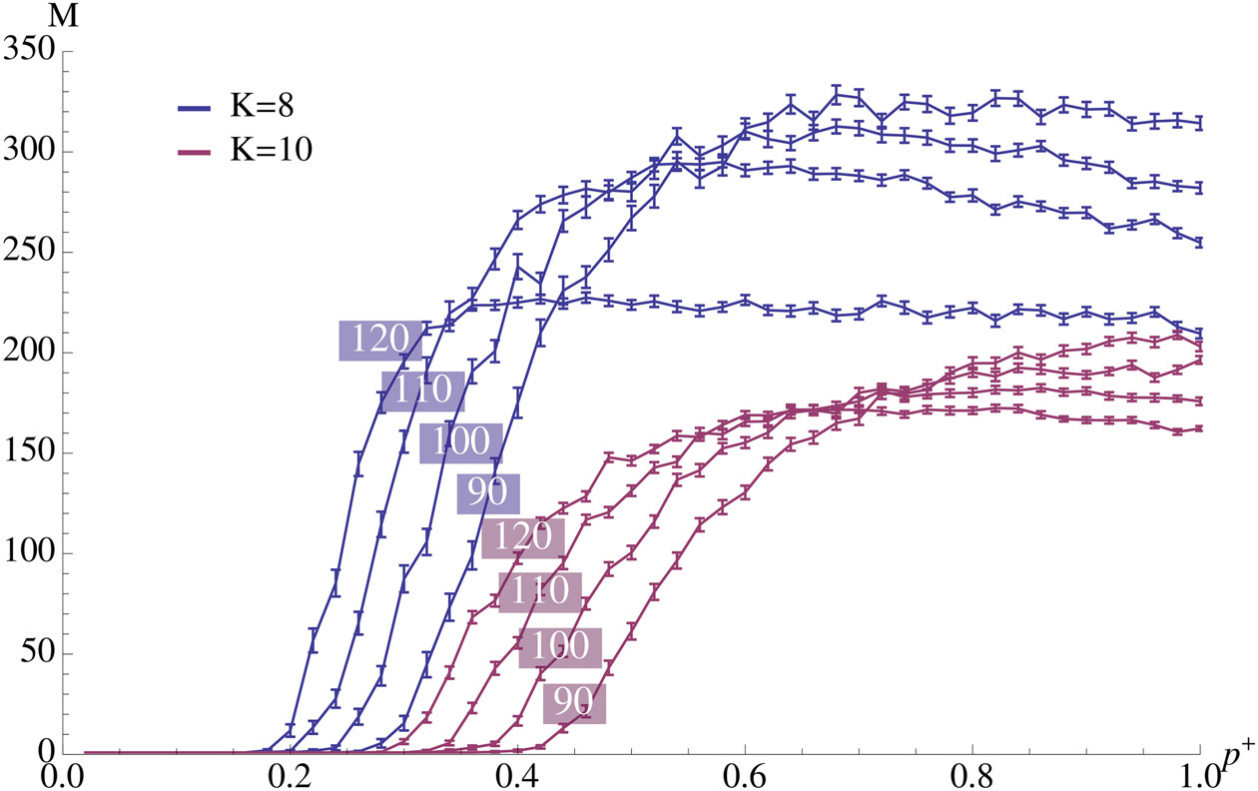
**Plasticity (afferent insertion probability, *p*^+^) vs. capacity for different pattern sizes and values of *K***. The relevant parameters are chosen as in Figure 2A where ρ_rec_*n* = 4. Error bars represent standard mean error and there are 100 trials per data point. We observe that values close to the optimum can be obtained with relatively small insertion probability. Note that the exact value of *p*^+^ is not very important as long as it exceeds a certain threshold.

**Figure 4:**
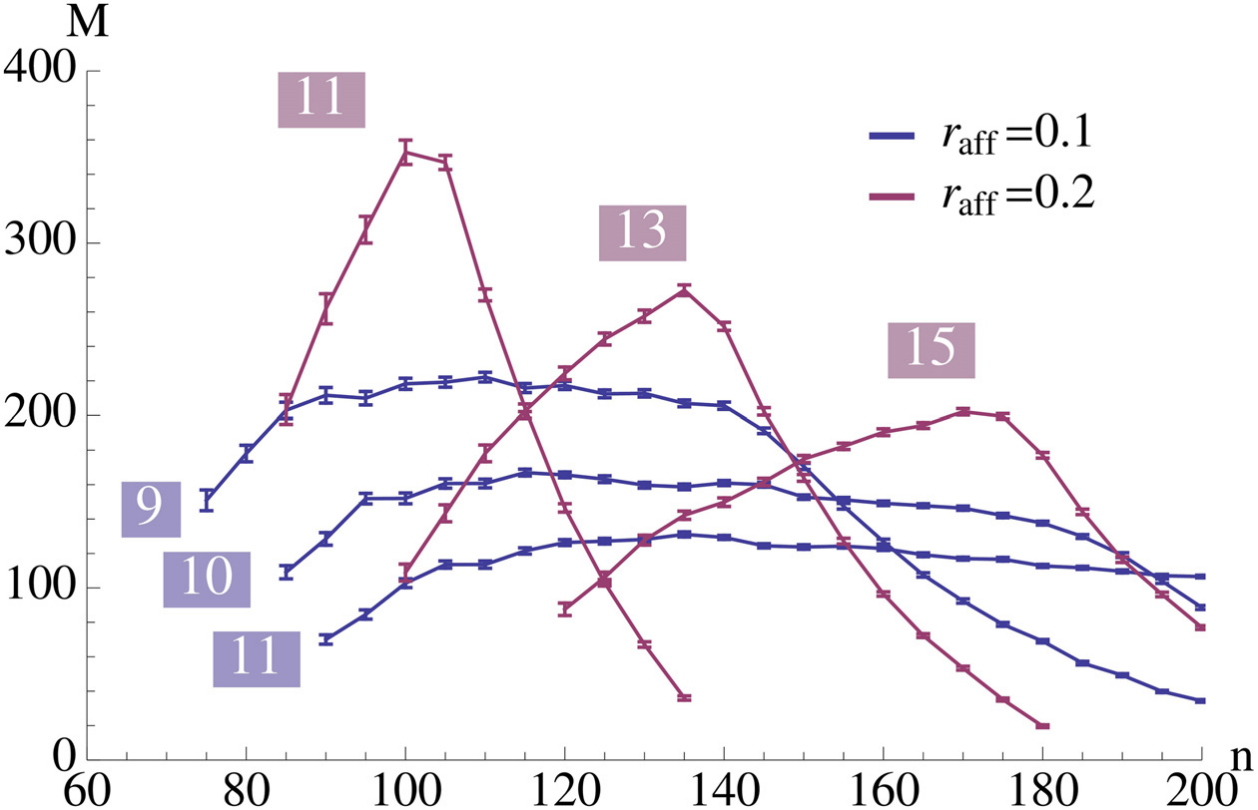
**This plot illustrates the same experiment as in Figure 2A, but we vary *r*_aff_ and provide the data for different values of *K* (as indicated in the plot)**. It shows that increasing *r*_aff_ can yield a higher capacity but it comes at the price of only working for a small range of possible pattern sizes. Each data point is the mean of 100 trials where capacity is only considered if at least 99% of the insertions were successful.

We analyze the model both mathematically and with simulations. The mathematical analysis investigates the limiting case *N* → ∞. Our main tool is the result of Janson et al. (2012) for bootstrap percolation in a random graph. We extend their result in order to analyze iterative retrieval of a pattern. As a side effect of our calculation we also deduce optimal parameters for a high memory capacity. In particular, we find that the desired plasticity has a non-trivial optimum: it should neither be too small nor too high, cf. Figure 3. Similarly, memory capacity depends on the patterns size in a unimodular way, cf. Figure 4, that is the pattern size should neither be too small nor too big.

## 2. Methods

### 2.1. Model overview and assumptions

#### 2.1.1. Setup and terminology

Let *G* be a directed graph with vertex set *V* = 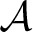 ∪ 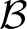 where the sets 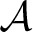 and 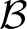 are of equal size *N* (cf. Figure 1). Edges between vertices of the same set are called *recurrent*, those from 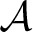 to 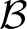 *afferent*. All edges between 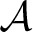 and 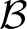 are directed toward 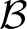. Edges can be either weak or strong. A vertex gets activated if it is connected to at least *K* active vertices by strong edges, where *K* is a parameter of the model.

We consider the following learning problem. Let (*A*_*i*_)_*i* ≥ 0_ and (*B*_*i*_)_*i* ≥ 0_ be sequences of random subsets (*patterns*) of 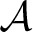 and 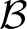, respectively, with sizes |*A*_*i*_| = |*B*_*i*_| = *n* for *i* ≥ 0. We sequentially present each pair (*A*_*i*_, *B*_*i*_) once. At the presentation of each pair we may change some of the afferent edges from strong to weak or vice versa. In the recall phase we activate all vertices of *A*_*i*_ and let activation propagate. The pair (*A*_*i*_, *B*_*i*_) is called *memorized* if activation of the vertices in *A*_*i*_ leads to an activation of the vertices in *B*_*i*_. More precisely, we want to activate at least α_fid_*n* vertices in *B*_*i*_ (fidelity), and at most α_spc_*n* vertices outside of *B*_*i*_ (specificity). An insertion is considered *successful* if the pair is memorized right after insertion.

Note that due to the presence of recurrent edges activation can *propagate*: a small set of initially active vertices in *B*_*i*_ (arising from activity in *A*_*i*_) can eventually activate a much bigger set. More precisely, we start with an active set consisting of the vertices in *A*_*i*_. In the first round we then activate all vertices that have *K* neighbors in *A*_*i*_ to which they are connected by strong edges. In the second round all vertices get activated that are connected by *K* strong edges to vertices in *A*_*i*_ *or* to vertices that were activated in the first round, and so forth. Note that here we tacitly assume that signal propagation is so fast that activation can take place in rounds. Since only strong edges count for activating a neuron, we define the *degree* deg(*v*; *S*) of a vertex *v* with respect to a set *S* ⊂ *V* to be the number of strong edges between *v* and *S*.

Observe that due to our setup the oldest associations have the worst quality. Moreover, we choose a pruning parameter (see below) in such a way that the expected number of strong edges remains constant regardless of the number of shown relations, i.e., the model is a palimpsest (see Nadal et al., 1986). (Note that we take the point of view that the edges within a pattern are fixed, while the afferent edges are plastic; that is, the model is a palimpsest for association learning, not for pattern learning.) We are thus interested in determining the maximum number *M* (the *capacity* of the model) of additional associations that can be learned so that the set *A*_0_ can still activate its partner *B*_0_.

We study learning in a sparse random setting. We assume that afferent edges are present with probability ρ_aff_, independently. Before learning starts we turn every afferent edge strong with probability *r*_aff_, independently. Note that *r*_aff_ impacts how many edges a vertex outside *B*_0_ receives from *A*_0_ which also depends on *n*.

As we assume that patterns *B*_*i*_ correspond to “concepts” that are already known, we insert recurrent edges as follows. Each edge in 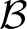 is present with probability ρ_rec_ independent of other edges and all of them are initially weak. For each pattern *B*_*i*_ we turn all the edges between pairs of vertices in it strong. In particular, 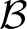 corresponds to a sparsely connected Willshaw network.

#### 2.1.2. Learning procedure

In order to learn an association (*A*_*i*_, *B*_*i*_) during its presentation we

- Turn each weak afferent edge between *A*_*i*_ and *B*_*i*_ strong (“insert it”) with probability *p*^+^,
- Turn each strong afferent edge between *A* \ *A*_*i*_ and *B*_*i*_ weak (“prune it”) with probability *p*^−^,

cf. Section 4.2 Note that the first step is a stochastic form of Hebbian learning (Barrows, 1998). The second step is a normalization step. Hence, we choose *p*^−^ in such a way that the expected degree for each vertex in *B*_*i*_ stays constant. Observe that the “randomness” assumption means that a vertex *b* ∈ *B*_*i*_ is expected to have ρ_aff_*n* edges from vertices in *A*_*i*_ out of which an *r*_aff_-fraction are strong and (*N* − *n*)ρ_aff_ edges from vertices in 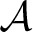 \ *A*_*i*_ out of which also an *r*_aff_-fraction are strong. The learning procedure will thus, in expectation, turn (1 − *r*_aff_)ρ_aff_*np*^+^ edges strong and *r*_aff_ρ_aff_(*N* − *n*)*p*^−^ edges weak. We thus set

(1) $$p^{-}=\frac{1-r_{\text{aff}}}{r_{\text{aff}}}\cdot \frac{n}{N-n}\cdot p^{+}.$$

### 2.2. Comparison with the model of Amit and Fusi

**Figure 5:**
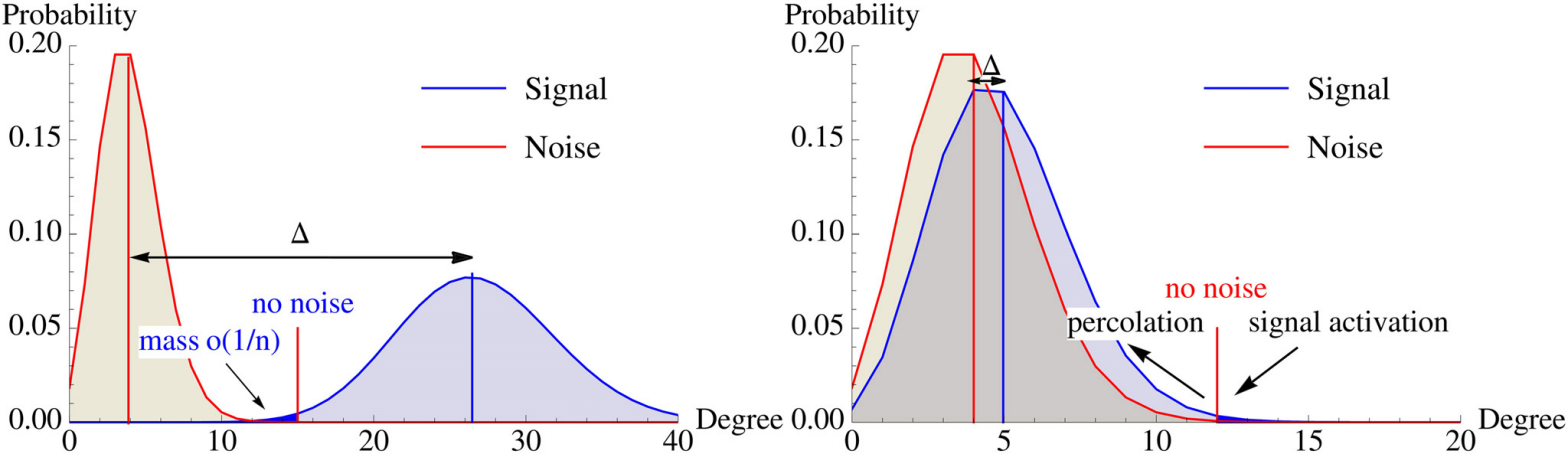
**Illustration of the degree distribution of vertices in 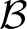 \ *B*_0_ (noise) and *B*_0_ (signal) w.r.t. *A*_0_**. **Left**: Without recurrent edges, the overlap of both distributions must be extremely small. **Right:** Percolation allows activating the pattern via recurrent edges with fewer strong afferent edges. This dramatically influences the location of the optimal activation threshold *K* and the signal degree distribution can be much closer to the noise distribution while the pattern is still memorized. The differences between both scenarios are exaggerated to highlight the different mechanisms.

Our model builds upon the work on the well-studied (Amit and Fusi, 1994) model (*AF model*) and its extensions (cf. Battaglia and Fusi, 1994; Brunel et al., 1998; Romani et al., 2008; Amit and Huang, 2010). In particular, the learning paradigm is identical. The main differences are:

- The AF model studies *auto-associative memory* instead of *hetero-associative memory*. Thus, it considers only one population of neurons (instead of two populations in our model), and in the learning phase patterns are presented instead of pairs of patterns. Consequently, the AF model does not need to distinguish between recurrent and afferent connections. It is well-known that association learning is easier to humans than pattern learning (see Fanselow, 1990).
- All studies on the AF model assumed a complete underlying graph. However, it is straightforward to extend the model to sparse, randomly connected graphs, cf. below. The asymptotics of the capacity remains the same; more precisely, both for the complete graph and the sparse random graph, it is possible to learn θ(*N*^2^/log^2^ *N*) patterns before the first pattern is forgotten. However, the density (probability of two neurons being connected by a synapse) will enter via the constant hidden in the θ-notation. Actually, it dramatically reduces the capacity for neuron populations of size, say, *N* = 5000, i.e., for magnitudes of *N* where neurophysiologically we may assume a constant density (cf. below).
- The AF model investigates whether an activated pattern forms an attractor state in the state space. Consequently, a pattern is remembered in the AF model if every neuron in a pattern *A* has at least *K* neighbors in *A*, and every neuron outside of *A* has less than *K* neighbors in *A*. This view is not suited if the underlying graph is assumed to be a sparse random graph, as there is always a constant probability that a vertex has less than *K* (strong or weak) neighbors in the pattern. A pattern containing such a vertex can then never be in an attractor state, even if all the edges in the pattern turn strong. We therefore require that only an α_fid_-fraction of the pattern is activated, where 0 < α_fid_ ≤ 1 is a parameter that we may choose. For α_fid_ = 1 we are back in the Amit-Fusi model.
  Note that the requirement in the AF model is weakest possible in terms of attractor networks. For example, one might ask what part of the state space is attracted into the pattern state. For such question, the update rule may be important, and it is known that iterative retrieval is superior to one-step-retrieval (Schwenker et al., 1996). However, all such questions break down if the pattern state is not a stable attractor.
- The other, and actually main, difference to the AF model is that we consider hetero-associative memory instead of auto-associative memory, i.e., we do not activate the pattern itself (and require that it stays active), but we activate a pattern *A*_*i*_ and investigate whether this pattern is able to activate its “partner” *B*_*i*_. Without recurrent edges this boils down to the question whether all (or, cf. above, an α_fid_-fraction) of the neurons in *B*_*i*_ have at least *K* neighbors in *A*_*i*_. This special case is equivalent to the question whether a pattern is memorized in the AF model. With recurrent edges propagation of activity will allow us to show that we actually need only a small fraction of the neurons in *B*_*i*_ to have at least *K* neighbors in *A*_*i*_; propagation of activity will then nevertheless ensure that an α_fid_-fraction of the neurons in *B*_*i*_ is activated (see Figure 5). In other words, the AF-model (or rather its hetero-associative equivalent) may be viewed as a starting point of our considerations, as we essentially copied the learning rule and also focus on fast learning. However, as we consider hetero-associative memory we are able to make use of recurrent edges.

Our assumptions are motivated by facts known from neurophysiology. We assume that the two neuronal ensembles are in different areas of the brain. A neuron in the brain is connected to 10–20% of its closest neighbors, and this number drops sharply with distance exceeding 200–300μ (see Song et al., 2005; Le Bé et al., 2006; Perin et al., 2011; Levy and Reyes, 2012). The size of the input layer of a cortical column contains roughly *N* ≈ 5000 neurons (Meyer et al., 2010). This is also roughly the number of neurons within a ball of radius 300 μ (Beaulieu and Colonnier, 1983). The data from Kalisman et al. (2005) suggest that plausible values for the densities within such neuron populations of such a size are of the order of 0.1–0.2, while the afferent density is substantially lower (Binzegger et al., 2004).

## 3. Results

### 3.1. Theoretical results

The effect of learning an association will diminish over time due to later pruning steps. Clearly, this is most critical for the association (*A*_0_, *B*_0_). In this section we thus analyze the recall properties of this association only. Note that we do not aim for precise asymptotics, but rather we give an intuition for the underlying mechanisms of the process. Within the calculations we will therefore make some simplifying assumptions (the Erdős-Rényi assumption in Section 3.1.2 and the Janson assumption in Section 3.1.4). In Section 3.1.5 we then discuss the effect of these assumptions. In order to study whether we can activate pattern *B*_0_ by activating *A*_0_ we need to know the degree distribution of vertices *b* ∈ *B*_0_ (for fidelity) and *b* ∈ *B* \ *B*_0_ (for specificity) into *A*_0_. To do so we first consider the probability that a single, fixed edge is strong.

#### 3.1.1. Edge probabilities

Let *a* ∈ *A*_0_ be arbitrary. For *b* ∈ 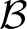 we denote by *p*_signal_ and *p*_noise_ the probability Pr[{*a, b*} strong | {*a, b*} is an edge] in the cases *b* ∈ *B*_0_ and *b* ∈ 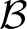 \ *B*_0_, respectively. First consider *b* ∈ 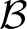 \ *B*_0_. After presentation of (*A*_0_, *B*_0_) we have *p*_noise_ = *r*_aff_ as the learning procedure did not touch the edge {*a, b*}. We show by straightforward induction that *p*_noise_ remains at this value regardless of how many additional pairs (*A*_*i*_, *B*_*i*_) are learned, so

(2) $$p_{\text{noise}}=r_{\text{aff}}$$

at any time. Indeed, after presenting one more association (*A*_*i*_, *B*_*i*_), {*a, b*} is strong with probability *r*_aff_ + (1 − *r*_aff_)*p*^+^ if *a* ∈ *A*_*i*_ (which happens with probability *n*/*N*) and with probability *r*_aff_ (1 − *p*^−^) if *a* ∉ *A*_*i*_ (which happens with probability (*N* − *n*)/*N*). Thus, Pr[{*a, b*}strong] = $\frac{n}{N}$ · (*r*_aff_ + (1 − *r*_aff_)*p*^+^) + $\frac{N-n}{N}$ · *r*_aff_ (1 − *p*^−^) = *r*_aff_ also in this case, where the last equality follows from Equation (1).

In contrast, *p*_signal_ changes after each association presentation. Let us denote by *p*_signal_(*i*) the value after *i* additional associations were learned. Then *p*_signal_(0) = *r*_aff_ + (1 − *r*_aff_)*p*^+^, and by considering an argument similar as above we see that with each new association the probability of an edge being strong drops as follows:

$$\begin{array}{l} p_{\text{signal}}(i+1)=\frac{N-n}{N}p_{\text{signal}}(i)+\frac{n^{2}}{N^{2}}\left(p_{\text{signal}}(i)+(p_{\text{signal}}(i))p^{+}\right)\\ \;+\frac{n(N-n)}{N^{2}}p_{\text{signal}}(i)(1-p^{-})\\ \;=p_{\text{signal}}(i)\left(1-\frac{n^{2}p^{+}}{N^{2}r_{\text{aff}}}\right)+\frac{n^{2}p^{+}}{N^{2}}, \end{array}$$

where the last inequality again follows from Equation (1). In particular, we find that the difference Δ(*i*):= *p*_signal_(*i*) − *r*_aff_ decays exponentially with *i*:

$$\begin{array}{l} \Delta (i+1)=p_{\text{signal}}(i+1)-r_{\text{aff}}\\ \;=p_{\text{signal}}(i)\left(1-\frac{n^{2}p^{+}}{N^{2}r_{\text{aff}}}\right)+\frac{n^{2}p^{+}}{N^{2}}-r_{\text{aff}}\\ \;=\left(p_{\text{signal}}(i)-r_{\text{aff}}\right)\left(1-\frac{n^{2}p^{+}}{N^{2}r_{\text{aff}}}\right)\\ \;=\Delta (i)\left(1-\frac{n^{2}p^{+}}{N^{2}r_{\text{aff}}}\right). \end{array}$$

Consequently, we obtain an explicit formula for *p*_signal_(*i*) as

(3) $$p_{\text{signal}}(i)=r_{\text{aff}}+\beta^{i}(1-r_{\text{aff}})p^{+},$$

where β:= 1 − (*n*/*N*)^2^ · *p*^+^/*r*_aff_. For short reference, we will denote by *p*_signal_ = *p*_signal_(*M*) the probability after *M* presentations, where *M* is the capacity of the system cf. below.

#### 3.1.2. Degree distribution

In order to investigate propagation of activity we need to know the degree distribution of vertices *b* ∈ 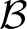 into *A*_0_. Assuming independence of the probabilities that we computed in the last section, we get

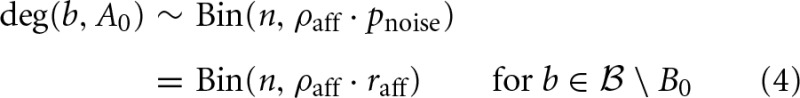



and

(5) $$\begin{array}{l} \text{deg}(b,A_{0})\sim \text{Bin}(n,\rho_{\text{aff}}\cdot p_{\text{signal}}(i))\\ \;=\text{Bin}(n,\rho_{\text{aff}}\cdot (r_{\text{aff}}+\beta^{i}(1-r_{\text{aff}})p^{+}))\text{ for }b\in {\text{B}}_{0}, \end{array}$$

and all these distributions are independent.

For all asymptotic computations we assume that the edges are independent.^1^ We call this the “Erdős-Rényi assumption,” since it implies that the edges between *A*_0_ and *B*_0_ are given by an Erdős-Rényi random bipartite graph model *B*_*n*,*n*;*p*_ for some edge probability *p*. Similarly, we assume that the edges between *A*_0_ and 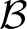 \ *B*_0_ and the edges within *B*_0_ are given by Erdős-Rényi random graphs *G*_*n*,*p′*_ (for some different edge probability *p*′). Under the “Erdős-Rényi assumption” Equations (4) and (5) are valid. Clearly, we do make some error here; however, one can show that the probability that the assumption is violated tends to zero for *N* tending to infinity. Similar results are known for the Willshaw model (Knoblauch, 2008). We abstain from estimating the error for finite *N*, but instead provide some experimental evidence in Section 3.2.

#### 3.1.3. Learning without recurrent edges

In order to understand the effect of recurrent edges, we first consider the case of no recurrent edges. This scenario is actually very closely related to the AF model. Recall that the AF model assumes that the input must be able to activate *all* neurons in the pattern (α_fid_ = 1.0). For a sparse setting this seems overly restricive. In this section we thus also consider the case α_fid_ = 0.5 (for which the calculations below are particularly easy). As we will see the benefits (in terms of memory capacity) of a such a seemingly much smaller value is in fact quite moderate.

In the previous section we argued that we may assume the degree distribution to be binomial. In this section we will furthermore assume that for large enough values binomial distributions are well approximated by normal distributions. Recall from Equation (3) that the expected probability for an edge between *A*_0_ and *B*_0_ to be strong is

$$p_{\text{signal}}=p_{\text{signal}}(i)=r_{\text{aff}}+\beta^{i}p_{\text{signal}}(0),$$

where *p*_signal_(0) is the probability immediately after presenting association (*A*_0_, *B*_0_), and $\beta =\left(1-\frac{n^{2}p^{+}}{N^{2}r_{\text{aff}}}\right)$. Recall also that the difference Δ(*i*) = *p*_signal_(*i*) − *r*_aff_ decays with each additional pattern by a factor of β.

The memory capacity *M* is determined by three variables: the factor β by which the differences Δ(*i*) decay, the initial difference Δ(0), and the minimal difference Δ for which the pattern can still be retrieved. More precisely, the capacity is given by *M* = log_β_ (Δ(0)/Δ).

As Amit and Fusi noticed in their seminal paper, in the *N* → ∞ limit it is possible to learn a large number of patterns by making the decay factor β very close to one. More precisely, setting *n* = θ(log *N*), the quotient Δ(0)/Δ turns out to be constant, and

(6) $$\begin{array}{l} M=\log_{1/\beta }(\Delta (0)/\Delta )=\frac{\log(\Delta (0)/\Delta )}{\log(1/\beta )}\\ \;=\theta \left(\frac{1}{\log(1/\beta )}\right)=\theta \left(\frac{N^{2}}{n^{2}}\right). \end{array}$$

Here we will investigate the effect of a smaller activity threshold α_fid_. The value of α_fid_ obviously does not change β and Δ(0). It only affects the minimal difference Δ. So we need to estimate Δ for various values of α_fid_.

The minimal difference Δ is determined by two requirements on the activation threshold *K*. Firstly, *K* must be large enough that no noise occurs. This is the case if the probability that a neuron in 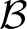 \ *B*_0_ has degree *K* is at most α_spc_*n*/*N*. Since we assume the degree distribution of such neurons to be binomially distributed with mean μ_spc_ = *n*ρ_aff_*p*_noise_ = *n*ρ_aff_*r*_aff_ and variance σ^2^_spc_ = *n*ρ_aff_*r*_aff_(1 − ρ_aff_*r*_aff_), we use the normal approximation of the binomial distribution to deduce that we need

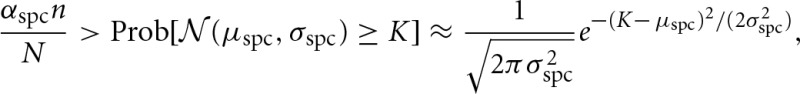



or equivalently

(7) $$K>n\rho_{\text{aff}}r_{\text{aff}}+\sigma_{\text{spc}}\sqrt{2\log\left(\frac{N}{\alpha_{\text{spc}}n\sqrt{2\pi \sigma_{\text{spc}}^{2}}}\right)}.$$

Secondly, *K* must be small enough that we can activate an α_fid_ fraction of *B*_0_. Similarly as above, this time using the normal distribution with mean μ_fid_ = *n*ρ_aff_*p*_signal_ and variance σ^2^_fid_ = *n*ρ_aff_*p*_signal_(1 − ρ_aff_*p*_signal_), we get for α_fid_ = 1 − $\frac{1}{n}$ that

(8) $$K<n\rho_{\text{aff}}p_{\text{signal}}-\sigma_{\text{fid}}\sqrt{2\log\left(\frac{1}{\alpha_{\text{fid}}\sqrt{2\pi \sigma_{\text{fid}}^{2}}}\right)}.$$

If, on the other hand, we are satisfied with α_fid_ = 0.5, then we only need the mean of the distribution to be larger than *K*, so we only need

(9) $$K<n\rho_{\text{aff}}p_{\text{signal}}$$

in this case.

For α_fid_ = 0.5 we may combine inequality Equation (7) and (9) to obtain an explicit formula for the minimal difference Δ = *p*_signal_ − *r*_aff_ that is sufficient for recall:

(10) $$\Delta \approx \frac{\sigma_{\text{spc}}}{n\rho_{\text{aff}}}\sqrt{2\log\left(\frac{N}{\alpha_{\text{spc}}n\sqrt{2\pi \sigma_{\text{spc}}^{2}}}\right)}.$$

Note that we need Δ < 1, as Δ is supposed to be a probability. From this we deduce that *n* cannot be too small. More precisely, we need *n* = Ω(log *N*), as already observed by Amit and Fusi (1994).

For α_fid_ = 1 − $\frac{1}{n}$, we may combine inequality Equation (7) and (8) to get a bound on Δ. In this case, an explicit solution is not possible. However, keeping in mind that Δ remains bounded as *N* → ∞, we may rewrite *p*_signal_ = *r*_aff_ + Δ to deduce

(11) $$\begin{array}{l} \Delta >\frac{\sigma_{\text{spc}}}{n\rho_{\text{aff}}}\sqrt{2\log\left(\frac{N}{\alpha_{\text{spc}}n\sqrt{2\pi \sigma_{\text{spc}}^{2}}}\right)}\\ \;+\frac{\sigma_{\text{fid}}}{n\rho_{\text{aff}}}\sqrt{2\log\left(\frac{1}{\alpha_{\text{fid}}\sqrt{2\pi \sigma_{\text{fid}}^{2}}}\right)}. \end{array}$$

Since σ_fid_ = θ($\sqrt{n}$) the second term tends to 0. On the other hand for *n* = θ(log *N*) the first term remains constant [and thus σ_spc_ = θ($\sqrt{\log N}$)]. Therefore, we will get the same asymptotic behavior for the memory capacity from Equations (10) and (11). Thus, in the limit we will not see any difference (not even in the leading constant factor). For small values of *n* and *N*, however, both terms in Equation (11) are of the same order of magnitude. So here we do see a difference between 100% activation and 50% activation. Note however that even if both terms are of the same order of magnitude we only gain a factor of ≈2—but we would gain much more if we could replace the plus sign in Equation (11) by a minus sign. Recurrent edges allow essentially that, as we will see in the next section.

#### 3.1.4. Learning with recurrent edges: percolation

In the previous section we derived that the number of patterns that can be learned satisfies $M\approx \frac{N^{2}r_{\text{aff}}}{n^{2}p^{+}}\log\left(\frac{\Delta (0)}{\Delta }\right)$, where Δ(0) = *p*_signal_(0) − *r*_aff_ is the difference between signal and noise at start and Δ is the minimal difference for which retrieval is possible. While this formula is asymptotically very satisfactory, it fails to give good results for realistic values like *N* = 5000 and *r*_aff_ = 0.1. Working out the numbers one sees that then the fraction $\frac{\Delta (0)}{\Delta }$ will be extremely close to 1 or even less than 1 (in which case no learning is possible at all). We have also seen that decreasing α_fid_ from 1.0 to, say, 0.5 has no dramatic effect as it only increases Δ by a factor of roughly two. Similarly, allowing more noise only increases Δ by a small, constant factor.

In this section we show that using recurrent edges and percolation theory can overcome this problem for small constants. Figure 5 illustrates the underlying idea. Without recurrent edges one has to ensure that the degree distributions of the signal and the noise are so far apart that one can choose an activation threshold *K* such that the noise distribution has only a tiny part to the left (as these are the vertices that will get activated outside the pattern), while the signal distribution should have a small part to the right of *K* (as these are the vertices within the pattern that will not get activated). Using iterative retrieval allows to essentially move the two distribution on top of each other, as the condition for the signal is replaced by “activate a small fraction” instead of “activate almost everything.”

Percolation or, more precisely, bootstrap percolation was studied by Janson et al. (2012) for random graphs. Given an Erdős-Rényi graph *G*_*n, p*_ and a random subset *A* of *active* vertices of size |*A*| = *a*. Percolation studies the question for which sizes of *A* (as a function of the size of the graph *n* and the edge density *p*) activity spreads to all (or at least almost all) vertices. Activity spreads according to a *K*-threshold rule, i.e., a vertex turns active if it has at least *K* active neighbors and once it turns active it remains active. Janson et al. (2012) gave a complete characterization of all occurring cases and phenomena. We do not state their result formally, but instead give a sketch of their proof. Subsequently, we then show how it can be transferred to our setting.

Let us recall the setup: we are given a random graph *G*_*n, p*_ and we start with a (random) subset *A* of size |*A*| = *a* of active vertices. Instead of immediately activating all vertices with enough active neighbors, we expose the random graph *G*_*n, p*_ step by step by the following, equivalent process.

Consider a set *U* of unexposed vertices and a set *E* of exposed vertices. At the beginning we initialize *U* with the vertices from *A* and let *E* start empty. Every time we expose a vertex from *U* (by removing it from *U*, adding it to *E* and exposing the edges from it to *V* \ *E*) we add newly active vertices to *U*. If *U* gets empty at some point in time we add a random (unexposed) vertex to it.

In order for the process to percolate one needs that at every time *t* there are still unexposed vertices, i.e., the set *U* is non-empty. Observe that at time *t* (that is, when |*E*| = *t*) every vertex in *V* \ *E* has revealed exactly *t* (potential) edges. That is, it is active at time *t* with probability *p*′ = Pr[Bin(*t, p*) ≥ *K*]. Let *S*(*t*) denote the set of vertices in *V* \ *A* that are active at time *t* and let *s*(*t*) = |*S*(*t*)|. Then *s*(*t*) is distributed as Bin(*n* − *a, p*′). Since we assume the process to percolate, the *t* exposed vertices are all active. Hence, the size of *U* at time *t* is *s*(*t*) + *a* − *t*.

So we percolate if and only if for all *t* we have Bin(*n* − *a, p*′) > *t* − *a*. In Janson et al. (2012), the authors proved that for large *n* we may replace the binomial distribution by its expectation (we call this the Janson assumption). Thus, we percolate if and only if we have

(12) (n−a)Pr[Bin(t,p)≥K]>t−a   for all t≥0.

Essentially, one can read off the conditions for percolation from Equation (12), cf. Janson et al. (2012) for the formal derivation. The key point is that whenever the edge probability is sufficiently high (e.g., *p* ≥ (1 + δ)log *n*/*n*, for any δ > 0) then we only need to activate a set *A* of size ≫ (*np*^*K*^)^−1/(*K*−1)^ = *o*(*n*) in order to have (almost) full percolation with high probability.

**Figure 6:**
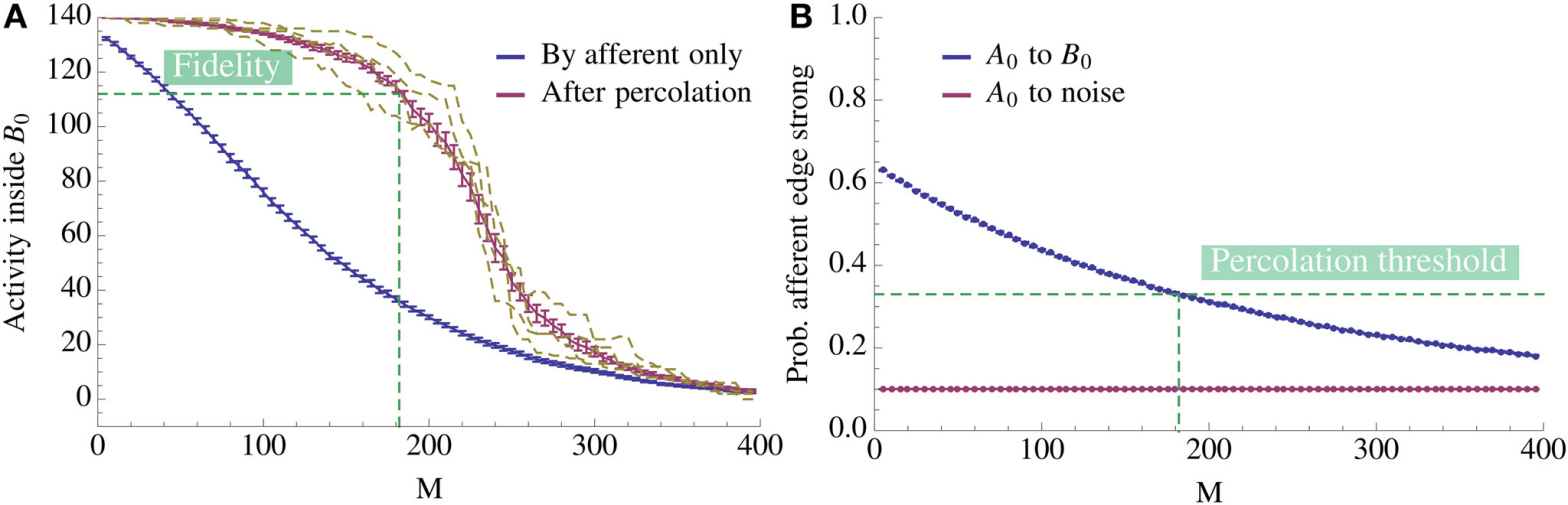
**Change of activity inside the pattern as more competing associations are learned**. **(A)** The *x*-axis denotes the number of associations exposed to the model and the y-axis gives the mean number of active vertices over 20 trials. We compare the activity due to afferent edges only (blue) and after percolation via recurrent edges (red). The dashed yellow curves give examples of single instances of the process after percolation. Note that since fidelity is 80% we count the pattern as being activated if we percolate to at least 112 vertices. Thus, in expectation we can learn 182 patterns for this choice of parameters. **(B)** Mean ratio of strong edges to all edges over 20 trials. We compare the density of strong edges amongst present edges between *A*_0_ and *B*_0_ and between *A*_0_ and 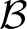 \ *B*_0_. As expected, due to normalization of strong edges the density toward 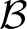 \ *B*_0_ remains fixed. Note also that the fidelity threshold from **(A)** induces a density threshold, dashed green line. Above threshold percolation works but below it does not. Here the value of the threshold is ≈ 0.33. For both plots *N* = 5000, *n* = 140, *K* = 12, *p*^+^ = 0.6, *r*_aff_ = 0.1, ρ_aff_ = 0.2, and the recurrent degree is 8. For both figures the error bars represent standard mean error.

We now transfer these results to the learning scenario studied in this paper. If we assume that within *B*_*i*_ the edges form a random graph with density ρ_rec_ then the above percolation result tells us that we only have to activate a tiny portion of *B*_*i*_ directly in order to achieve full activation of *B*_*i*_. Figure 6 illustrates this effect. As Figure 6A shows, it suffices to activate even a small bootstrap afferently in order to activate the whole pattern by percolation. Moreover, observe the threshold effect: while the afferent density stays above some threshold value, percolation activates almost the complete pattern; below this threshold, activity does not spread. This is the basis for our analysis: once we know the threshold, we can compute how the afferent density evolves over time to determine when it hits the threshold (Figure 6B).

It remains to determine the threshold. Actually, our situation is even better than the one studied in Janson et al. (2012): every vertex in *B*_*i*_ has an afferent degree into *A*_*i*_ distributed as Bin(*n*, ρ_aff_*p*_signal_). For some vertices this degree will be at least *K* and they thus get activated immediately. Other may have degree almost *K*, but the recurrent edges to the vertices that were activated immediately will bring the degree above *K*, etc. For a formal study we proceed similarly as above: we consider a set *U* of unexposed vertices that at *t* = 0 contains all vertices whose afferent degree is at least *K*. While percolation runs we again add active vertices to *U*. Observe that in this scenario a vertex of *B*_*i*_ is active at time *t* with probability

$$\begin{array}{l} p^{\prime }=\mathrm{Pr}[\text{Bin}(n,\rho_{\text{aff}}p_{\text{signal}})+\text{Bin}(t,\rho_{\text{rec}})\geq K]\\ \;=\mathrm{Pr}[\text{Bin}(n,\rho_{\text{aff}}p_{\text{signal}})\geq K]\\ \;+\sum_{i=0}^{K-1}\mathrm{Pr}[\text{Bin}(n,\rho_{\text{aff}}p_{\text{signal}})=i]\cdot \mathrm{Pr}[\text{Bin}(t,\rho_{\text{rec}})\geq K-i]. \end{array}$$

Again we denote by *S*(*t*) the set of vertices in *B*_*i*_ that are active at time *t* and let *s*(*t*) = |*S*(*t*)|. Then *s*(*t*) is distributed as Bin(*n, p*′). In order to percolate we need *s*(*t*) > *t* for all 0 ≤ *t* < *n*. Replacing the binomial distribution by its expectation (as we may do under the Erdős-Rényi assumption by Janson et al., 2012) we obtain that we percolate if and only if

(13) $$n\cdot \mathrm{Pr}[\text{Bin}(n,\rho_{\text{aff}}p_{\text{signal}})+\text{Bin}(t,\rho_{\text{rec}})\geq K]>t\;\text{for all }t\geq \text{0}.$$

For a fixed value of ρ_rec_ Equation (11) thus allows us to determine the probability of edges being strong afferently *p*_signal_ that we need in order to achieve percolation.

We close this section with the remark that while percolation has a dramatic effect for finite values, it does not change the asymptotics of the memory capacity. To see this observe that we need to be able to activate at least one vertex in *B*_*i*_ due to the afferent edges alone. By a similar argument as for Equation (11), we thus get

(14) $$\begin{array}{l} \Delta_{\text{percolation}}>\frac{\sigma_{\text{spc}}}{n\rho_{\text{aff}}}\sqrt{2\log\left(\frac{N}{\alpha_{\text{spc}}n\sqrt{2\pi \sigma_{\text{spc}}^{2}}}\right)}\\ \;-\frac{\sigma_{\text{fid}}}{n\rho_{\text{aff}}}\sqrt{2\log\left(\frac{n}{\sqrt{2\pi \sigma_{\text{fid}}^{2}}}\right)}. \end{array}$$

Note that the main change compared to Equation (11) is the sign of the second term. As before, for *N* → ∞, the first term will remain constant [for *n* = θ(log *N*)], while the second term will tend to 0. Hence, we will not see any difference in the asymptotic capacity. The influence of percolation is limited to finite values of *N*; but, as we saw in Figure 4, the differences of the two models are substantial for values of *N* and ρ_aff_ as they occur in the brain.

#### 3.1.5. Error estimates

The calculations in the previous sections rely on some approximations that are all valid in the *N* → ∞ limit. There are three sources of errors that need consideration:

1. The Erdős-Rényi assumption (independence) may not hold.
2. The Janson assumption [cf. Equations (12) and (13)] may not hold.
3. There is an error term that comes from replacing the binomial distribution by a normal approximation.

In the previous section we handled (1) and (2) by arguing that in the limit the probability that at least one these properties does not hold tends to zero. By then analyzing the situation under the condition that the Janson assumption and the Erdős-Rényi assumption do hold, cf. Equation (12), we get an estimate for what happens in the “typical” case. Unfortunately, to actually quantify the errors seems very hard, as for example, the paper Janson et al. (2012) does not provide precise bounds for the probability that (2) is violated.

In this section we thus show experimentally that the errors induced by the approximations (1)–(3) are indeed small for the chosen parameters. For each K, **Figure 9** contains four curves:

- The simulation result;
- We use simulations to estimate the threshold for percolation *p*_signal_ in an Erdős-Rényi random graph, and computed the capacity by Equation (6);
- We use the Janson assumption in Equation (13) for *t* = 0, …, α_fid_*n* to estimate *p*_signal_, and compute the capacity by Equation (6);
- We use Equation (13) to estimate *p*_signal_ as in (b), but with the binomial distributions replaced by normal approximations. Then we compute again the capacity by Equation (6).

The four curves quantify the errors 1–3 in the following way:

- In (b) we use the Erdős-Rényi assumption, but nothing else. So the difference between a and b quantifies the error of type 1.
- In (c) we use the Erdős-Rényi assumption and the Janson assumption. So the difference between b and c quantifies the error of type 2.
- In (d) we use the Erdős-Rényi assumption, the Janson assumption, and the normal approximations. So the difference between c and d quantifies the error of type 3. Finally, the difference between a and d quantifies the overall contribution of all three error sources.

To compare the errors to the second order terms in Equations (11) and (14), recall that these terms are at least the difference between the capacities with and without recurrent edges, up to error terms of type 1, 2, and 3. Therefore, we also plotted the capacity without recurrent edges for different K (including the K that maximizes the capacity). It is clearly visible that the difference between the capacity with recurrent edges (highest blue curve) and the capacity without recurrent edges (highest violett curve) is much larger than the error terms. Thus, the errors of type 1, 2, and 3 are small for plausible parameter values.

#### 3.1.6. The optimal plasticity constant

From our consideration, we can derive the optimal value for the plasticity *p*^+^. Note first that the minimal difference Δ = *p*_signal_ −*r*_aff_ for which we can still recall is independent of *p*^+^, regardless of α and regardless of whether we use percolation. Since the capacity is

(15) $$\begin{array}{l} M=\frac{\log(\Delta (0)/\Delta )}{\log(1/\beta )}=\log\left(\frac{(1-r_{\text{aff}})p^{+}}{\Delta }\right)\frac{1}{\log(1/\beta )}\\ \;\approx \log\left(\frac{(1-r_{\text{aff}})p^{+}}{\Delta }\right)\left(\frac{N^{2}r_{\text{aff}}}{n^{2}p^{+}}\right), \end{array}$$

we essentially need to maximize a function of the form log(*c*_1_*p*^+^) · (*c*_2_/*p*^+^). Such a function takes its maximum at *p*^+^ = *e*/*c*_1_, where *e* = 2.718…. Hence, the optimal *p*^+^ is

$$p^{+}=\frac{e\Delta }{1-r_{\text{aff}}}.$$

For the case without recurrent edges this resembles the findings in Romani et al. (2008). For the maximal capacity we hence get

(16) $$\begin{array}{l} M\approx \log\left(\frac{(1-r_{\text{aff}})p^{+}}{\Delta }\right)\left(\frac{N^{2}r_{\text{aff}}}{n^{2}p^{+}}\right)\\ \;=\frac{N^{2}r_{\text{aff}}(1-r_{\text{aff}})}{n^{2}e\Delta }. \end{array}$$

Note that *M* is not independent of ρ_aff_ since Δ ~ 1/$\sqrt{\rho_{\text{aff}}}$.

#### 3.1.7. Noise tolerance

We study two types of noise tolerance, so called *query noise*, where the activation of *A*_0_ is imperfect and *recurrent noise*, where we start the recall with active vertices in 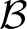 \ *B*_0_.

In the case of query noise we activate *A*_0_ with λ precision, λ ∈ [0, 1], meaning that we activate λ*n* vertices chosen u.a.r. from *A*_0_ and (1 − λ)*n* vertices chosen u.a.r. from 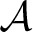 \ *A*_0_. Note that since there are *n* vertices active in 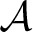 at the start of percolation every vertex in 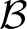 \ *B*_0_ expects the same amount of inputs as if *A*_0_ was activated with precision λ = 1 so the specificity constraint is unaffected. However, for vertices in *B*_0_ they now expect to receive λ*np*_signal_ + (1 − λ)*nr*_aff_ signals from 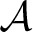. We thus have that we can still recall *B*_0_ after *i* insertions of competing associations if

$$\lambda (p_{\text{signal}}(i)-r_{\text{aff}})>\Delta .$$

One easily checks that the difference in capacity between precision 1 and precision λ is

$$\log_{1/\beta }(1/\lambda )=\theta \left(\frac{N^{2}\log(1/\lambda )}{n^{2}}\right).$$

**Figure 7:**
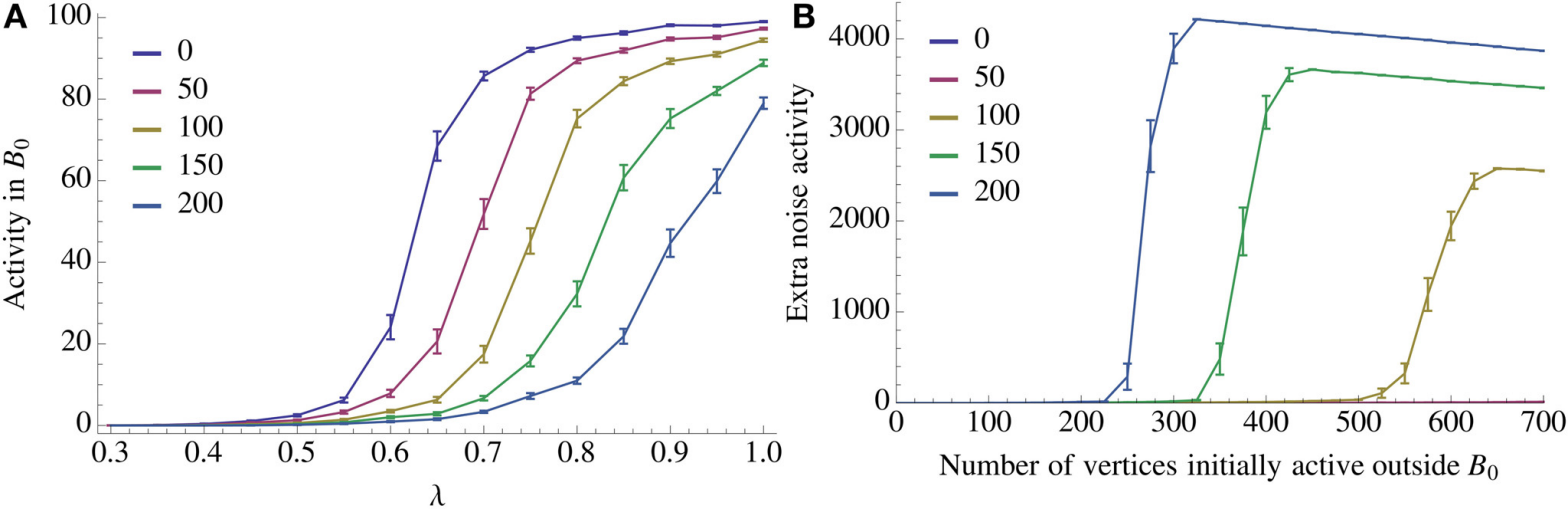
**Resilience of recall against noise**. We look at two types of noise, an imperfect activation of *A*_0_ (query noise, **A**), and interference within 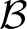 \ *B*_0_ (recurrent noise, **B**). In the case of query noise we u.a.r. activate λ*n* vertices in *A*_0_ and (1 − λ)*n* vertices in 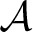 \ *A*_0_. We observe a threshold in λ for recall which depends on the number of inserted competing associations. A further discussion of the query noise can be found in Section 3.1.7. For recurrent noise we activate together with *A*_0_ a fixed amount of vertices in 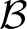 \ *B*_0_. For recurrent noise we activate together with *A*_0_ a fixed amount of vertices chosen u.a.r. in 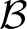 \ *B*_0_. We also observe a threshold in how many such vertices the model can tolerate which depends on the number of patterns stored recurrently within 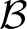. For both figures the parameters of the process are the same as in Figure 2A with *n* = 100, *K* = 12, and ρ_rec_*n* = 8, and error bars represent standard mean error.

For recurrent noise with *m* noisy vertices the bootstrap consists of *A*_0_ and *m* vertices chosen u.a.r. from 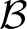 \ *B*_0_. In this case the activation of *B*_0_ w.r.t. the fidelity requirement is not affected but we run the risk of percolation within 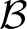. Note that the edges within 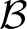 are not independently strong so we cannot directly apply the percolation theory for Erdős-Rényi graphs. However, empirical observations (see Figure 7B) indicate that there is still a threshold phenomenon occurring for percolation which depends on the number of patterns stored in 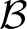. Moreover, the same figure shows that the capacity of the system is extremely stable against recurrent noise.

### 3.2. Experimental results

The theoretical results obtained in Section 3.1 are for the limiting case *N* → ∞. It is not possible to obtain explicit error terms since the error terms for the threshold density *p*_signal_ in the bootstrap percolation are not known explicitly. For this reason we test our results in a bioplausible range with *N* = 5000 neurons in 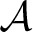 and 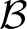 each (cf. Section 2.2).

For all the relevant figures we perform one shot learning as described in Section 2.1.2. In order to realize a recurrent density of ρ_rec_ within the patterns we proceed as follows: we initialize the set 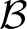 as a random graph with edge probability ρ_rec_ with all the edges weak. When we insert a pattern in 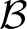 we turn all the edges inside the pattern strong. In that way we inherit the density of ρ_rec_ for each pattern from the global density within 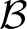.

Figure 2 demonstrates how memory capacity depends on the pattern size *n* when all parameters of the process are fixed and chosen in some optimal way, as argued below. The capacity is the expected number of associations which can be inserted until the first association cannot be recalled any more (due to pruning and/or noise). Throughout we chose α_fid_ = 0.8 and α_spc_ = 1.0 as parameters for fidelity and specificity.

In general, a fixed set of parameters ρ_aff_, *r*_aff_, ρ_rec_, *p*^+^, and *K* will only work for a finite range of values for *n*: if *n* is too large, then noise is too large and the specificity criterion is violated. On the other hand, if *n* is too small, then we will not be able to satisfy the fidelity condition even immediately after learning.

In the following figures we illustrate the connections between the various parameters for the case ρ_aff_ = 0.2. In the figures we only show data points for which reliability was at least 99%, meaning that in 99% of the cases the first association could be recalled before competing associations were inserted.

**Figure 8:**
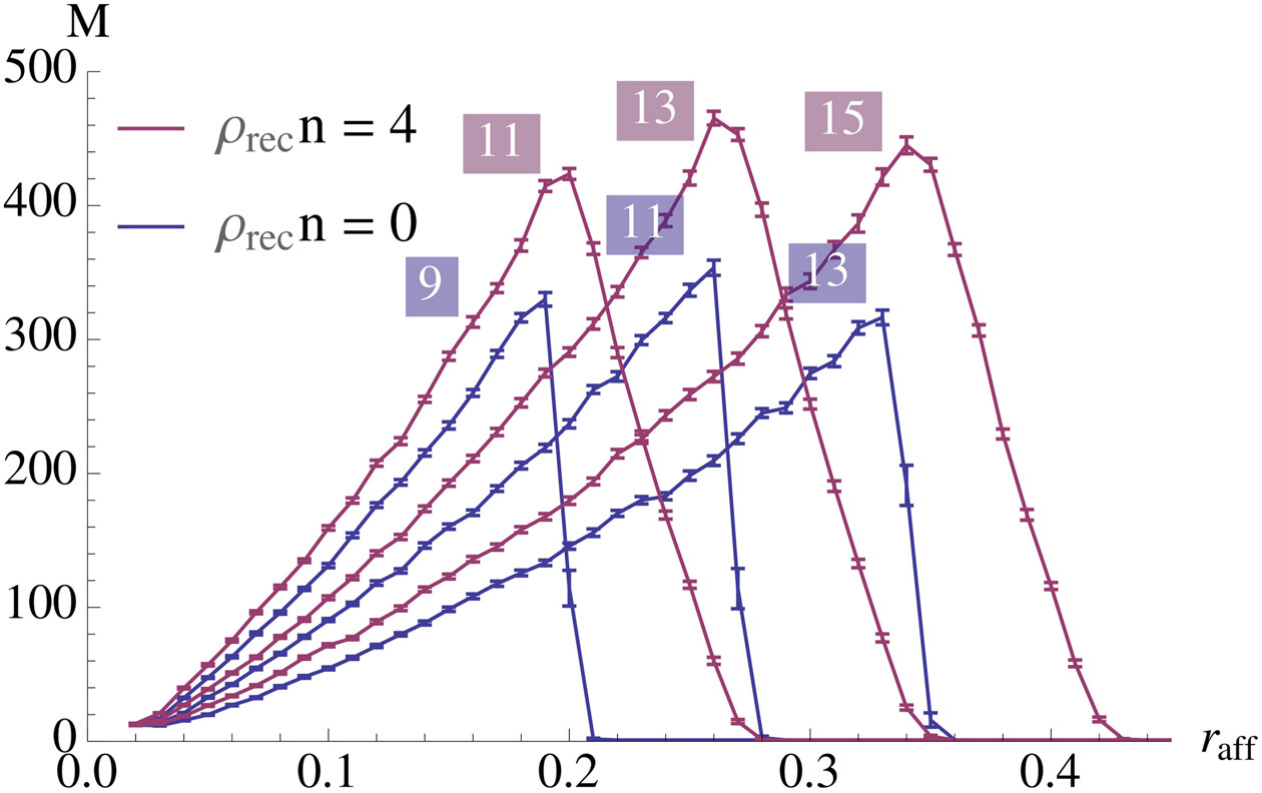
**Strong edge density (probability of afferent edges being strong, *r*_aff_) vs. capacity for *n* = 100**. The labels on the curves denote which value of *K* was used to generate it. The blue curve for *K* = 11 and red curve for *K* = 13 maximize capacity. Here *N* = 5000, ρ_aff_ = 0.2, and *p*^+^ = 1. Each data point is the mean of 100 samples and the error bars represent standard mean error.

Figure 8 demonstrates the effect of varying the probability of afferent edges being strong, i.e., *r*_aff_, for a fixed value of *n* (here *n* = 100). As it turns out, for each *K* the curve is unimodular and the maximal values of these curves are also unimodular. The figure shows the best *K* for recurrent degree 0 respectively 4. It is worthwhile to note that for fixed *K* the curves drop sharply if *r*_aff_ exceeds a certain value (as then noise takes over). However, for ρ_rec_ > 0 this drop is less dramatic, making the setup more stable.

Figure 8 seems to indicate that a value of *r*_aff_ ≈ 0.25 is a good choice. In order to test that we compared in Figure 4 the effect of *r*_aff_. We found that while for larger *r*_aff_ the maximum value that we can achieve is indeed higher, this comes at the price of robustness. More precisely, for larger values of *r*_aff_ the curves (for a fixed *K*), tend to be very pointed, while for smaller *r*_aff_ we can have plateaus with almost the same value. This is the reason for our choice of *r*_aff_ = 0.1 in Figure 2.

Figure 3 illustrates our choice of *p*^+^ = 0.6. We see that when the remaining parameters are fixed, we essentially get a threshold phenomenon: *p*^+^ needs to be sufficiently large, but a further increase does not have a positive effect any more (but may even decrease performance). Intuitively, this phenomenon occurs because percolation within 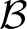 becomes possible with a bootstrap of size *n* before the association is forgotten afferently. A further increase of *p*^+^ thus only increases this effect and therefore does not increase the learning capacity.

Now we are ready to explain our choice of parameters for Figure 2A: we chose *r*_aff_ = 0.1 and *p*^+^ = 0.6, as suggested by Figures 3, 8. The figure on the right side of Figure 2 shows a similar plot for ρ_aff_ = 0.05. Here it turned out that *r*_aff_ = 0.05 yields better results (due to the smaller memory capacity), so we chose this value, and the learning probability is again *p*^+^ = 0.6. For both cases, and each expected recurrent degree, *K* was chosen so that we obtain stable results for *n* in a wide range. In the case of zero recurrent degree, the sparsity enforces a small value of *K* to allow learning at all; in turn, this means that no value of *K* works for a large interval, so we chose *K* = 3 which yields the best (even though still quite small) capacity for large *n*'s.

Figure 7 demonstrates the two types of noise tolerance we study. In the case of query noise, Figure 7A, we choose our parameters as in Figure 2A with *n* = 100, *K* = 12, and ρ_rec_*n* = 8. In this setting the model is able to satisfy the fidelity requirement with λ = 0.7 and even after 100 insertions of competing associations the relation (*A*_0_, *B*_0_) can still tolerate λ = 0.8. For the recurrent noise we observe that with only a few patterns stored recurrently in 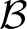 the model does not react to recurrent noise at all. This happens because either the necessary bootstrap size for percolation is too large or percolation within 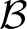 is simply impossible due to the density of strong recurrent edges being too low. However, once sufficiently many patterns have been inserted in 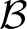 percolation becomes possible and we observe a threshold behavior, see Figure 7B.

**Figure 9:**
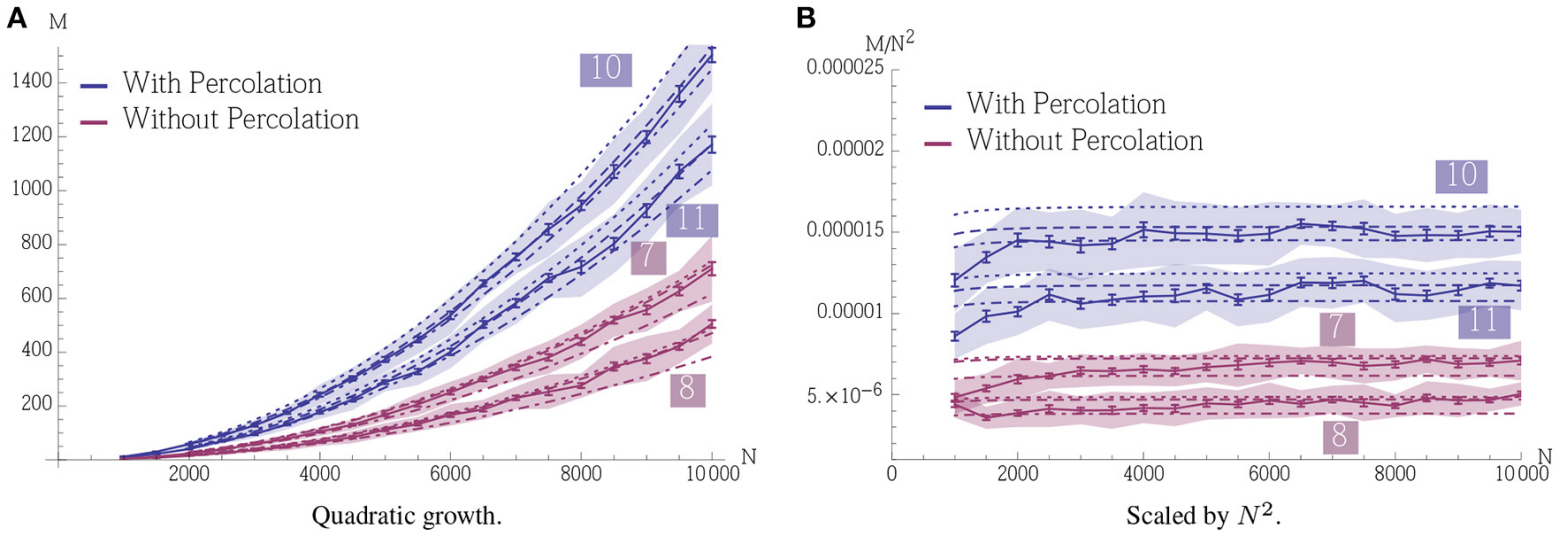
**Demonstration of quadratic growth and comparison between simulation and theoretical predictions (A). (B)** displays the same data but scaled by *N*^2^ to estimate the constant of the leading term. In case of the simulation, whole curve, the error bars represent standard mean error for 25 trials and the envelope represents the standard deviation. The dashed curve, (b), is calculated using a theoretical prediction where first Δ_percolation_ is estimated using a simulation. The simulation determines the smallest *p*_signal_ for which percolation is still possible for a pattern pair in an Erdős-Rényi random graph and the estimator is an average over 500 such trials. The dashed curve is then obtained using Equation (6). The dotted curve, (c), is also calculated using a theoretical prediction. There we estimate Δ_percolation_ using Equation (12) such that it is satisfied for *t* = 0, …, α_fid_*n* and use Equation (6) again to obtain the curve. Finally the dot-dashed curve, (d), is the prediction where Δ_percolation_ is obtained by replacing the binomial distributions in Equation (12) by normal distributions. For a further discussion on these predictions, b, c, and d, and their importance we refer the reader to Section 3.1.5. All parameters in this figure are fixed except for *N*, they are the same as in Figure 2A with *n* = 100 and ρ_rec_*n* = 8. Note that for a fixed *K* the growth stops when the specificity constraint becomes violated due to afferent noise. However, this can be accounted for by altering the parameters, we refer to Section 3.1.3 for a further discussion.

Figure 9 gives an example of quadratic growth with three theoretical predictions for comparison that quantify the different approximations made in the theoretical predictions, cf. Section 3.1.5.

## 4. Discussion

### 4.1. Model assumptions

### 4.1.1. Synapses and learning

The synapses in our model only have two states: they are either weak or strong. The learning rule follows the Hebbian paradigm “fire together, wire together,” followed by a normalization step. Learning mechanisms in the brain are more complicated. In particular, for spike-timing dependent plasticity (STDP) the timing of pre- and post-synaptic spike is crucial. However, it has been shown by Abbott and Nelson (2000); Gerstner and Kistler (2002a,b) that STDP resembles Hebbian learning when there is no systematic time shift between different inputs, and at the same time it normalizes the input of each neuron (see Kempter et al., 1999; Abbott and Nelson, 2000; Song et al., 2000; Abbott and Gerstner, 2004; Gilson et al., 2010).

The question whether synapses are binary is unsettled and vividly disputed in Graupner and Brunel (2010); Barbour et al. (2007); Satel et al. (2009). However, some STDP experiments indicate that synapses in the hippocampus are indeed binary: synapses that have been potentiated by an STDP protocol can not be potentiated a second time, but can be depressed again, and vice versa as in Petersen et al. (1998); O'Connor et al. (2005). Also, while such experiments last for minutes, the change is sudden and strong (a factor of 2–3, see Petersen et al., 1998; O'Connor et al., 2005). These findings are compatible with our assumptions of stochastic Hebbian learning.

### 4.1.2. Activity and dynamics

It is well-known that the brain encodes some information in the firing rate of neurons, and many computational papers take this point of view (e.g., Amit and Fusi, 1994). However, there are also other ways the brain encodes and processes information. E.g., when humans are asked to discriminate between pictures of animals and non-animals, then task-related eye-saccades can be observed after 120 ms (Kirchner and Thorpe, 2006). This amazing speed indicates that feedback loops or rate based encoding do not play a role for these ultra-fast processes, since each region in the brain has only 10–20 ms to process and transmit the signal. Thus, it seems that at least some type of hypothesis forming is done in a single feed-forward sweep of information, based on one or only very few spikes per neuron. Various other physiologic and psychologic experiments came to similar conclusions (Thorpe and Imbert, 1989; Allison et al., 1999; Liu et al., 2002; Crouzet et al., 2010; 't Hart et al., 2013, see also Johnson and Olshausen, 2002 for a review). We designed our model to fit a sweep of activity as described above, and thus we only count whether a neuron emits at least one spike, ignoring any further spikes of this neuron. Janson et al. (2012) proved that such a sweep is extremely fast: For a pattern with *n* vertices it takes at most time *O*(log log *n*) if the transmission delays of all edges is 1. In our context *n* = *O*(log *N*), so percolation only needs time *O*(log log log *N*). If the transmission delays are drawn from an exponential distribution with mean 1, then Einarsson et al. (2014) showed that the sweep is even faster: it takes at most constant time, independent of *n*.

## 4.2. Pattern sizes and plasticity

Our simulations show that stochastic Hebbian learning enables sparsely connected neuronal ensembles to perform one shot association learning. There is a tradeoff between reliability and capacity. For smaller pattern sizes the successfully inserted patterns can be memorized for a long time yielding a large expected capacity. However, a large portion of the insertions for small patterns are not successful, even with their optimal plasticity parameter *p*^+^ = 1. For larger patterns the optimum *p*^+^ is <1 and every pattern is stored successfully but the capacity drops proportional to $\frac{N^{2}}{n^{2}}$. By keeping *n* fixed and varying the plasticity parameter we have a similar tradeoff: if plasticity is too small associations are poorly stored in the first place but if it is too large the ongoing activity in the network will rapidly overwrite older associations. For a fixed population size *N* the optimum plasticity parameter decays proportional to $\frac{1}{\sqrt{n}}$. Since the growth rate of the capacity is quadratic we have that eventually every neuron will take part in multiple associations. This turns out to be the case even for *N* = 5000 in a sparsely connected network.

### Conflict of interest statement

The authors declare that the research was conducted in the absence of any commercial or financial relationships that could be construed as a potential conflict of interest.

## Footnotes

^1^Strictly speaking, edge probabilities are *not* independent. For example, if there exists 1 ≤ *i* ≤ *M, a*_1_, *a*_2_ ∈ *A*_0_ ∩ *A*_*i*_ and *b* ∈ *B*_0_ ∩ *B*_*i*_ then the events that {*a*_1_, *b*} is strong respectively that {*a*_2_, *b*} is strong are positively correlated.
